# Hospital staff reports of coworker positive and unprofessional behaviours across eight hospitals: who reports what about whom?

**DOI:** 10.1136/bmjoq-2023-002413

**Published:** 2023-11-14

**Authors:** Rachel Urwin, Antoinette Pavithra, Ryan D McMullan, Kate Churruca, Erwin Loh, Carolyn Moore, Ling Li, Johanna I Westbrook

**Affiliations:** 1 Australian Institute of Health Innovation, Macquarie University, Sydney, New South Wales, Australia; 2 St Vincent's Health Australia Ltd Fitzroy, Fitzroy, Victoria, Australia

**Keywords:** Communication, Healthcare quality improvement, Health services research, Nurses, Patient safety

## Abstract

**Background:**

Workplace behaviours of healthcare staff impact patient safety, staff well-being and organisational outcomes. A whole-of-hospital culture change programme, Ethos, was implemented by St. Vincent’s Health Australia across eight hospitals. Ethos includes a secure online submission system that allows staff across all professional groups to report positive (Feedback for Recognition) and negative (Feedback for Reflection) coworker behaviours. We analysed these submissions to determine patterns and rates of submissions and identify the coworker behaviours reported.

**Method:**

All Ethos submissions between 2017 and 2020 were deidentified and analysed. Submissions include structured data elements (eg, professional role of the reporter and subjects, event and report dates) and a narrative account of the event and coworker behaviours. Descriptive statistics were calculated to assess use and reporting patterns. Coding of the content of submissions was performed to classify types of reported coworker behaviours.

**Results:**

There were a total of 2504 Ethos submissions, including 1194 (47.7%) Recognition and 1310 (52.3%) Reflection submissions. Use of the submission tool was highest among nurses (20.14 submissions/100 nursing staff) and lowest among non-clinical services staff (5.07/100 non-clinical services staff). Nurses were most frequently the subject of Recognition submissions (7.56/100 nurses) while management and administrative staff were the least (4.25/100 staff). Frequently reported positive coworker behaviours were non-technical skills (79.3%, N=947); values-driven behaviours (72.5%, N=866); and actions that enhanced patient care (51.3%, N=612). Medical staff were the most frequent subjects of Reflection submissions (12.59/100 medical staff), and non-clinical services staff the least (4.53/100 staff). Overall, the most frequently reported unprofessional behaviours were being rude (53.8%, N=705); humiliating or ridiculing others (26%, N=346); and ignoring others’ opinions (24.6%, N=322).

**Conclusion:**

Hospital staff across all professional groups used the Ethos messaging system to report both positive and negative coworker behaviours. High rates of Recognition submissions demonstrate a strong desire of staff to reward and encourage positive workplace behaviours, highlighting the importance of culture change programmes which emphasise these behaviours. The unprofessional behaviours identified in submissions are consistent with behaviours previously reported in surveys of hospital staff, suggesting that submissions are a reliable indicator of staff experiences.

**WHAT IS ALREADY KNOWN ON THIS TOPIC**

- Unprofessional behaviour in hospitals negatively impacts patient safety, staff well-being and workplace culture.

**WHAT THIS STUDY ADDS**

- Descriptions of positive and unprofessional coworker behaviours that relate to communication, collaboration and teamwork are prevalent among staff submissions to a hospital online messaging system. Staff made submissions about coworkers within and outside of their own professional groups illustrating the interactive, collaborative nature of healthcare settings.

**HOW THIS STUDY MIGHT AFFECT RESEARCH, PRACTICE OR POLICY**

- Hospital professional accountability and culture change programmes should use whole-of-hospital strategies, rather than focus on individual professional groups. Opportunities should be provided to acknowledge positive behaviours by coworkers as well as report unprofessional behaviours.

## Introduction

Healthcare settings that demonstrate positive workplace and organisational cultures are associated with improved patient outcomes.^1^ From an organisational perspective, the positive impact of an engaged workforce includes lower absenteeism and stress,^2^ and decreased burn-out.^3^ Moreover, well-supported and motivated healthcare workers provide better quality of care to patients with fewer errors, and lower infection and mortality rates.^2 4^ An important component of a positive workplace for healthcare staff is an environment where employee voice is facilitated and speaking up about concerns related to coworker or patient safety is encouraged.^5 6^ Previous studies have also demonstrated that when strong norms for constructive confrontation exist, the negative impacts or consequences of workplace conflict are tempered, and team-based decision-making can be improved.^7^

In contrast, negative or unprofessional behaviours among healthcare workers undermine teamwork and communication, impacting not only staff well-being, retention, job satisfaction and commitment to the organisation,^8 9^ but also clinical performance and patient outcomes.^10^ Unprofessional behaviours range from subtle behaviours such as lack of responsiveness, passive aggression and incivility to more overtly hostile, bullying and inappropriate behaviours such as physical and verbal abuse.^10^ These behaviours are highly prevalent in healthcare.^11–13^ Randomised simulation trials exploring the effect of one of the most prevalent behaviours, being spoken to rudely, have shown adverse consequences with reduced team performance and patient safety.^14–16^

Interventions to foster positive workplace culture and reduce unprofessional behaviours in healthcare settings include professional accountability programmes,^17–20^ promotion of speaking up behaviours that give voice to concerns about coworker behaviours and safety,^5 21 22^ and staff recognition programmes which encourage peer recognition and reinforcement of positive behaviours.^3 23^

The Co-worker Observation Reporting System (CORS) programme is part of a professional accountability programme developed by the Center for Patient and Professional Advocacy at Vanderbilt University Medical Center to promote professionalism among primarily physicians by allowing coworkers to report unprofessional behaviours. Non-punitive feedback is provided to reported staff about their workplace behaviour from peer messengers.^13 17 24^ An evaluation of CORS in use at three US hospitals reported 71% of physicians who received peer messenger feedback had no subsequent reports in the following year.^24^ Qualitative content analysis of 120 randomly selected CORS reports found that the behaviours described could be organised into four domains: competent medical care; clear and respectful communication; integrity and responsibility, and that most reports described disrespectful or offensive communication.^25^ A recent study in three US medical centres demonstrated that the CORS process could also be effectively implemented for staff nurses.^26^

Programmes that increase accountability are designed to influence individual, interprofessional and organisational dynamics and normalise positive behaviours which consequently reduce unprofessional behaviours and improve outcomes.^3 27^ Theories of behaviour change highlight the value of empowerment approaches along with amelioration of negative behaviours.^28^ Positive feedback increases self-efficacy and intrinsic motivation^29^ and expressions of gratitude benefit both the recipient and the reporter.^30^ Supplementing accountability programmes with opportunities to provide positive feedback,^31^ and the promotion of active role modelling of positive behaviours^32^ are demonstrated to improve a range of interpersonal, professional and organisational outcomes.^2 31 33 34^

Built on the principle that all staff and patients should feel welcome, valued and safe, St Vincent’s Health Australia (SVHA) developed the Ethos programme, a whole of hospital professional accountability and culture change programme.^11 19 35 36^ The programme comprises several elements, including training staff to identify and speak up directly about unprofessional behaviours, as well as the recognition and reward of positive staff behaviours, while aiming to respond quickly and consistently to staff behaviours that fall below the expectations of the organisation.^11 19 35 36^ A key component of the Ethos programme is an online messaging system, which allows all staff to submit messages related to both positive and unprofessional behaviours of coworkers. Submissions may be made anonymously, providing an opportunity to raise concerns when staff do not feel safe or able to address the behaviour with their coworker directly. Staff who are recognised for positive behaviour receive direct manager feedback; positive reports may also be incorporated into the facility ‘rewards and recognition’ programme, where available.^19^ Submissions describing incidents of behaviour that undermine staff and patient safety are triaged and non-punitive feedback delivered by trained peer-messengers to provide an opportunity for staff to reflect on their behaviour.^19 37^ Serious unprofessional behaviours, for example, assault are managed via formal, traditional disciplinary processes. A mixed-methods study was conducted to evaluate the effectiveness of the Ethos programme to reduce unprofessional behaviours.^19 36–40^ A survey conducted prior to the introduction of Ethos revealed baseline information on the prevalence, type and impact of unprofessional behaviours among staff at SVHA hospitals.^41 42^ In this study, we investigated the utilisation of the online messaging system by staff at eight hospitals. We aimed to determine patterns and rates of submissions by professional group and identify the types of coworker behaviours reported.

## Methods

### Ethos messaging system

The Ethos programme was implemented between 2017 and 2020 through staged introductions across eight hospitals in Victoria, New South Wales and Queensland.^19 36^ The online messaging system allows all hospital staff to make submissions about coworker behaviour that either promotes or undermines patient or staff safety: Feedback for Recognition submission (‘Recognition’; reporting behaviour that positively impacts workplace culture); or Feedback for Reflection submission (‘Reflection’; identifying behaviour that negatively impacts workplace culture). For all submissions the user, who can opt to remain anonymous, provides information including: their professional group; the date and location of the incident; the name and professional group of the person who is the subject of the submission; a narrative description of the event, including who was involved and the context. There is no word limit for event descriptions.

### Deidentified Ethos submission data

All Ethos submissions made between July 2017 and December 2020 were deidentified by a designated hospital employee before the data were provided to the research team. Deidentification was to protect the privacy of the users who made submissions and the subjects of submissions. The research team were not able to account for multiple submissions by the same person or multiple submissions about the same subject. Deidentified data were further cleaned by the research team to eliminate duplications and unusable or incomplete submissions, such as reports with all incident details missing.

### Coding scheme development

To categorise the behaviours described in Ethos submissions, narrative incident descriptions that were included in each submission were analysed using inductive and deductive methods.^43^

Feedback for Recognition submissions: A classification for positive workplace behaviours in healthcare was developed by drawing on hospital, programme and professional bodies' code of conduct manuals^44–50^ and literature^51–58^ which outline positive professional characteristics. Using an inductive, iterative process of review and discussion among the research team, positive behaviours and attributes described in Recognition submissions were grouped into seven categories:

1. Patient care delivery and performance (eg, treatment and care effectiveness, safety, manner and quality of care, patient-centredness and timeliness).
2. Notable non-technical skills (eg, teamwork, coordination, collaboration, communication, other).
3. Notable technical skills (eg, extensive knowledge across area of expertise/practice, demonstrated excellence, skill and competence under pressure, complex project management, problem-solving and critical thinking).
4. Overcompensating for gaps (eg, in resources, task, skill shortages through personal effort and time).
5. Values-driven behaviours (eg, ethical, compassionate, empathetic, supportive, respectful, affording others dignity).
6. Advocacy for other people (eg, serving as a mediator, speaking up for patient care/dignity/security/safety, offering patients additional supports or linkage to support services, demonstrating support towards colleagues and advocating for staff needs).
7. Leadership, teaching or coaching skills (eg, demonstrating decision-making, organisational skills, role-modelling positive behaviour and technical/non-technical practice and skills, mentoring and teaching other staff required knowledge and skills to perform their roles, creating and environment of psychosocial safety where other staff feel welcome, learn and able to perform well).

Feedback for Reflection submissions: Reflection submissions were deductively analysed using an existing classification of 26 unprofessional behaviours that form the basis of the LION (**L**ongitudinal **I**nvestigation **O**f **N**egative behaviour) survey.^41^ The LION survey was used to assess the prevalence and types of unprofessional behaviours prior to Ethos implementation.^41^ These behaviours encompass a spectrum ranging from rudeness to physical assault. Any submissions that did not include at least one of the 26 behaviours were grouped together as ‘other’ and were later categorised using a grounded theory-guided approach.

**Figure 1:**
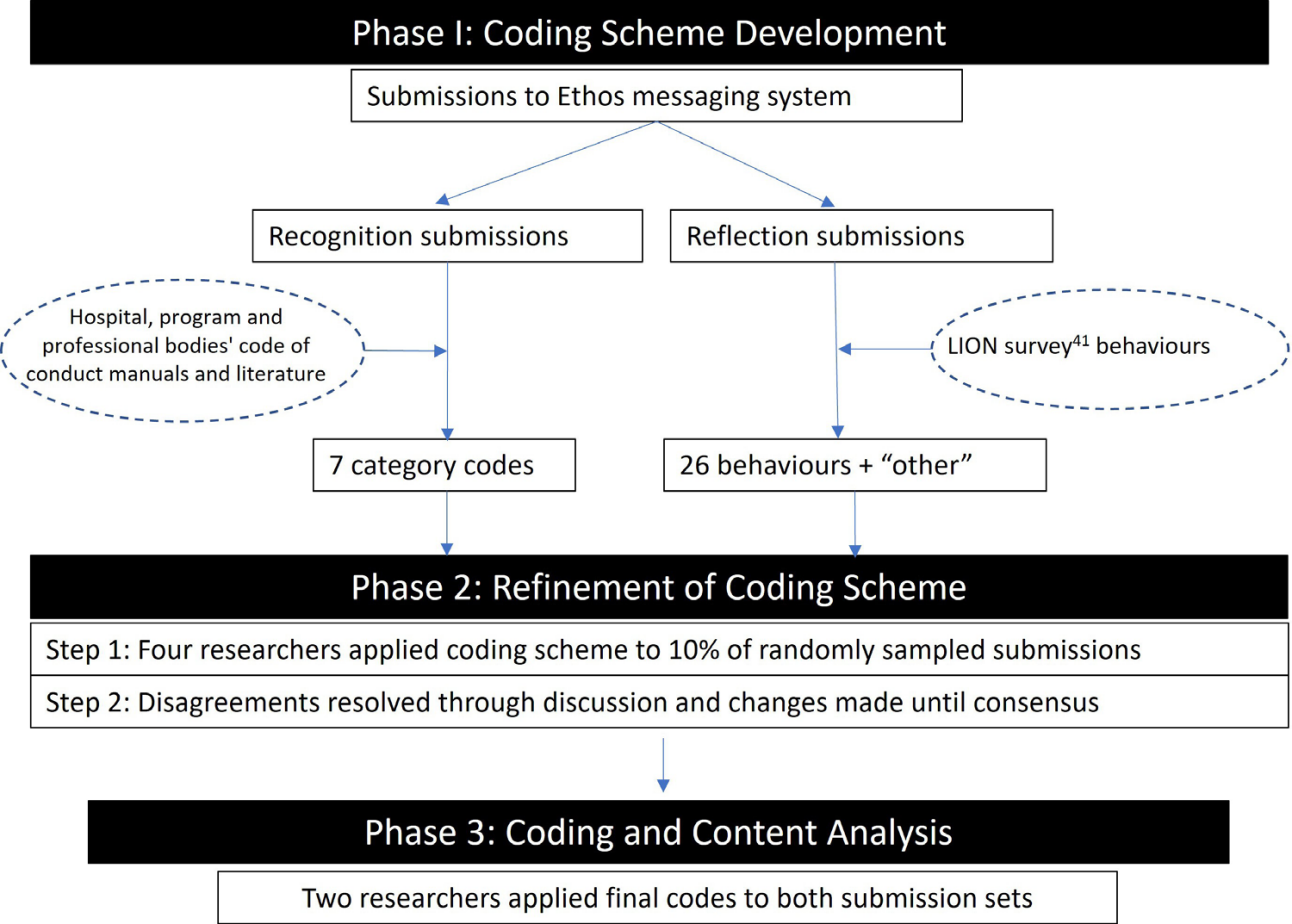
Methodology for data preparation and coding of Ethos submissions. LION: Longitudinal Investigation Of Negative behaviour.

Four researchers coded 10% of all Recognition and Reflection submissions and any disagreements or uncertainty were resolved through discussion. Once consensus was achieved one researcher (RU) coded feedback for Recognition submissions while another researcher (AP) coded all remaining feedback for Reflection submissions (figure 1).

### Statistical data analysis

To compare usage and reporting patterns of the Ethos reporting tool by professional groups, we calculated the rate of submissions per 100 staff for each profession, and by type of submission, that is, Recognition or Reflection. Related 95% Wald CIs were calculated. Similar calculations were conducted for the subject of submissions (ie, who the submission is about) per 100 staff, for each professional group.

Radar plots were used to show the patterns of submissions within (ie, intraprofessional reporting) and across professional groups (ie, interprofessional reporting) by submission type. Frequency and proportion of positive workplace behaviours reported in Recognition submissions and unprofessional behaviours reported in Reflection submissions were calculated.

### Patient and public involvement

Patients or the public were not involved in the design, or conduct, or reporting, or dissemination plans of our research.

## Results

### Use of the Ethos reporting tool 2017–2020

A total of 2504 Ethos submissions (14.06 submissions per 100 staff) were made across eight hospitals between July 2017 and December 2020, including 1194 (47.7%) Recognition and 1310 (52.3%) Reflection submissions. The median time between an event and a submission was 1 day (IQR: 0–5). A preliminary analysis showed some variations in patterns of use across sites and over time. However, large differences in hospital size, duration of the programme at each hospital, and other factors including the COVID-19 pandemic prevented a detailed analysis of programme utilisation over time.

Ethos submission rates (table 1) were highest among nurses (20.14 total submissions/100 nurses, 95% CI 19.27 to 21.05), who used the tool at similar rates for both Recognition (9.99 submissions/100 nurses, 95% CI 9.35 to 10.68) and Reflection submissions (10.15/100 nurses, 95% CI 9.5 to 10.84). Non-clinical services staff had the lowest rate of submissions (5.07/100 non-clinical services staff, 95% CI 4.34 to 5.93) and made twice as many Reflection submissions (3.44/100 staff, 95% CI 2.84 to 4.17) compared with Recognition submissions (1.63/100 staff, 95% CI 1.23 to 2.16).

**Table 1:** Users and subjects of Ethos submissions by professional group, 2017–2020, across eight hospitals

	Feedback for Recognition submissions N (submissions per 100 staff; 95% CI)	Feedback for Reflection submissions N (submissions per 100 staff; 95% CI)	Total submissions N (submissions per 100 staff; 95% CI)
Users of the Ethos reporting tool by professional group			
Nursing	787 (9.99; 9.35 to 10.68)	799 (10.15; 9.5 to 10.84)	1586 (20.14; 19.27 to 21.05)
Medical	99 (3.38; 2.78 to 4.1)	118 (4.03; 3.37 to 4.8)	217 (7.4; 6.51 to 8.41)
Allied health and clinical services	169 (7.39; 6.39 to 8.55)	169 (7.39; 6.39 to 8.55)	338 (14.79; 13.4 to 16.31)
Non-clinical services	48 (1.63; 1.23 to 2.16)	101 (3.44; 2.84 to 4.17)	149 (5.07; 4.34 to 5.93)
Management and administrative	87 (5.00; 4.07 to 6.13)	121 (6.95; 5.85 to 8.25)	208 (11.95; 10.52 to 13.57)
Total	1190* (6.7; 6.34 to 7.07)	1308† (7.36; 6.99 to 7.76)	2498 (14.06; 13.56 to 14.58)
Subject of Ethos submissions by professional group			
Nursing	595 (7.56; 6.99 to 8.16)	538 (6.83; 6.3 to 7.41)	1133 (14.39; 13.63 to 15.18)
Medical	202 (6.89; 6.03 to 7.87)	369 (12.59; 11.44 to 13.85)	571 (19.48; 18.1 to 20.97)
Allied health and clinical services	148 (6.47; 5.54 to 7.57)	139 (6.08; 5.18 to 7.14)	287 (12.55; 11.27 to 13.99)
Non-clinical services	175 (5.96; 5.16 to 6.88)	133 (4.53; 3.84 to 5.35)	308 (10.49; 9.44 to 11.66)
Management and administrative	74 (4.25; 3.4 to 5.3)	129 (7.41; 6.28 to 8.75)	203 (11.66; 10.25 to 13.27)
Total	1194 (6.72; 6.36 to 7.1)	1308‡ (7.36; 6.99 to 7.76)	2502 (14.08; 13.58 to 14.6)

*Four submissions made by ‘other’ staff (ie, volunteers, students) not included.

†Two submissions made by ‘other’ staff (ie, volunteers, students) not included.

‡Two submissions made about volunteers not included.

### Subjects of Ethos submissions

Overall, nurses were the most frequent subject of all submissions (N=1133, 45.2%, table 1). Nurses were the subject of more Recognition submissions compared with other professional groups (7.56/100 nurses, 95% CI 6.99 to 8.16). Medical staff were the subject of more Reflection submissions (12.59/100 medical staff, 95% CI 11.44 to 13.85).

### Intraprofessional and interprofessional reporting

**Figure 2:**
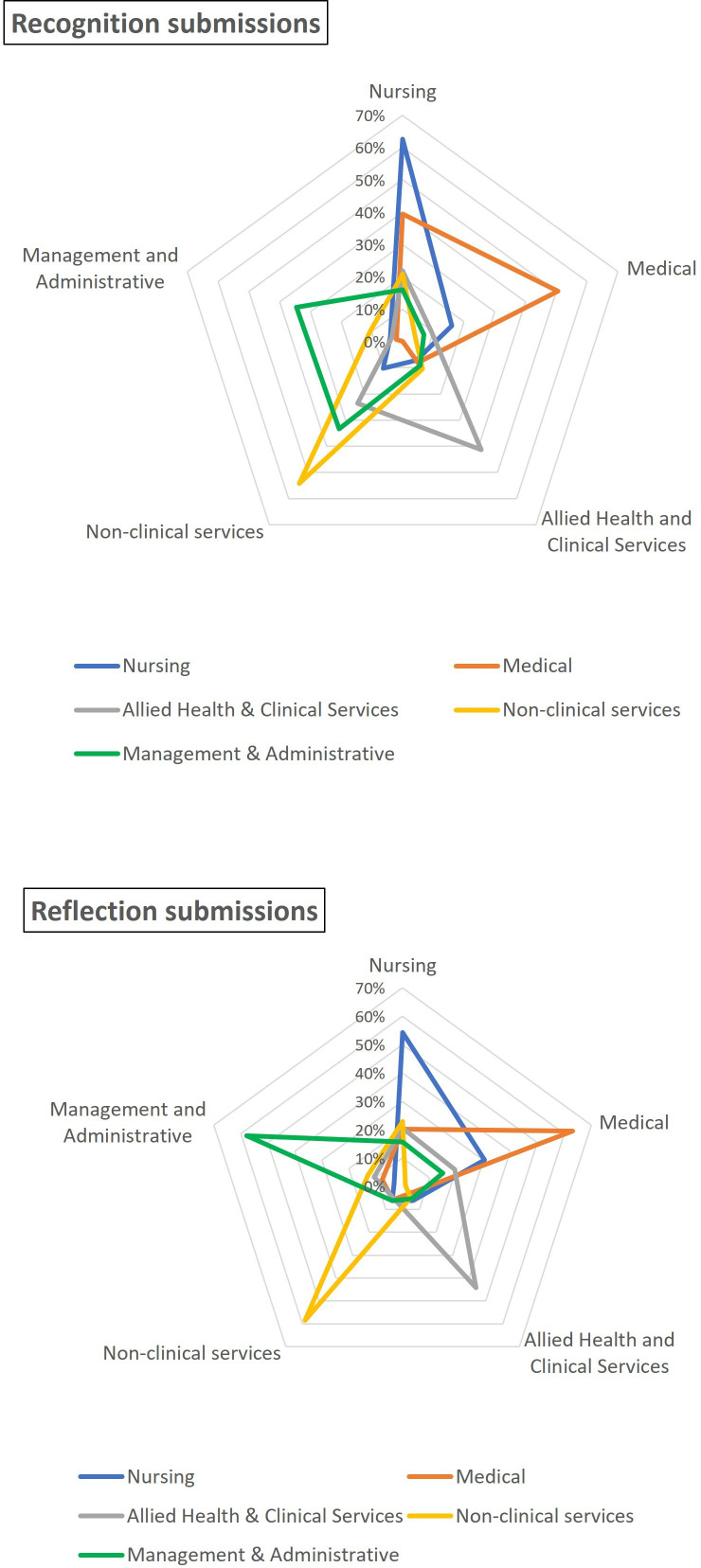
Radar plots illustrating the patterns of interprofessional and intraprofessional reporting for Recognition submissions and Reflection submissions.

Intraprofessional reporting, where the author and subject of the submission were from the same professional group, occurred in 54.4% (n=1361) of all submissions. Interprofessional reporting comprised 45.6% (n=1143) of submissions. Intraprofessional reporting was most frequent across all professional groups (figure 2 and online supplemental tables 1 and 2). For example, medical staff were the subjects of 50.5% of Recognition submissions and 63% of Reflection submissions made by other medical staff.

10.1136/bmjoq-2023-002413.supp1 Supplementary data

Interprofessional reporting varied by professional group. For example, nurses were the subject of 39.4% of Recognition and 20.2% of Reflection submissions made by medical staff, while non-clinical services staff were the subject of no Recognition and 5.9% of Reflection submissions made by medical staff. Most Reflection submissions made by management and administration staff (57.4%) were about other management and administration staff (57.4%), however, only 34.5% of their Recognition submissions were intraprofessional, with almost equal Recognition submissions (33.3%) about non-clinical services staff.

### Positive behaviours described in Recognition submissions

A range of clinical settings, scenarios and positive behaviours were described in Recognition submissions (table 2), from brief acknowledgement of a colleague’s workplace-enhancing attitude ‘*[deleted] is consistently friendly and personable with both staff and patients. [deleted] always makes me smile’* to lengthier reports of clinical excellence and compassionate care leading to enhanced patient and staff experience ‘*[deleted] showed great patience and resilience as he was doing a difficult [procedure]. [deleted] treated the patient with respect and answered all questions. It is so good to see someone treat another human being in such a positive way. [deleted] always gives his patients time and understanding and he is lovely to work with’*. More than one behaviour may have been included in a single submission.

**Table 2:** Illustrative examples of the seven categories of positive behaviours identified in Ethos feedback for Recognition submissions

Categories of positive behaviours	No of reports (%)	Illustrative examples		
		Event description	Profession of positive agent	Profession of submitter
Notable non-technical skills	947 (79.3)	“(deleted) has consistently been a wonderful team player in (deleted), especially during the COVID transitions of wards. He always has a positive attitude and always willing to go above and beyond to help his colleagues. (deleted) has excellent communication with us, which in turn allows us to provide the best care for our patients”	Pharmacy	Pharmacy
Values-driven behaviour	866 (72.5)	“(deleted) is such a compassionate nurse towards all patients and colleagues. (deleted) had a (deleted) family who were frustrated and upset at (deleted) due to a misunderstanding, and was able to deescalate the situation while making the family at ease and provide support. She goes out of her way to provide genuine empathy and care to patients and families.”	Nurse	Nurse
Patient care delivery and performance	612 (51.3)	“(deleted) has assisted me with a number of patients within the hospital. (deleted) has always offered to assist with patients, particularly who are CALD patients and are often quite vulnerable due to language barriers. (deleted) has always delivered patient centred care with kindness and respect. (deleted) is a very thoughtful nurse who always going above and beyond to help me whenever she can with patients. (deleted) is an amazing nurse and a true player”	Nurse	Allied Health
Leadership, teaching or coaching skills	231 (19.4)	“As a trainee I had a very difficult morning… (deleted) was incredibly supportive. He gave advice and constructive feedback. He was reassuring and allowed me time to make some notes and offered me the opportunity to repeat a practice examination. As a supervisor, (deleted) went over and above to help make a difficult day much easier”	Medical	Trainee (Medical)
Overcompensating for gaps	97 (8.1)	“Stepped up when short of staff, helped out in kitchen and laundry”	Allied Health	Allied Health
Advocacy for other people	74 (6.2)	“(deleted) came in to the hospital yesterday evening, despite not being on call, to advocate for a critically unwell (deleted) patient under the (deleted) that she knew well from a (deleted). She … accompanied the patient to theatre in the morning to explain the patient’s complex medical history to the treating doctors”	Medical	Medical
Notable technical skills	45 (3.8)	“My colleagues and I had exhausted all options with other support teams, until our case was escalated to (deleted). (deleted) was calm and patient over the phone, but was also extremely helpful and knowledgeable. He resolved our technical issue just before we were about to give up. (deleted) should be commended for his amazing work.”	Other non-clinical	Allied Health

Notable non-technical skills were most frequently described (N=947, 79.3%). Values-driven behaviours were highlighted in 866 (72.5%) submissions, while behaviours that enhance patient care were described in 612 (51.3%) submissions. The phrase ‘above and beyond’ was used to describe the behaviour of staff in 107 (9%) Recognition submissions.

### Unprofessional behaviours described in Reflection submissions

Table 3 summarises the frequency of 26 unprofessional behaviours reported in Reflection submissions. More than one behaviour may have been included in a single submission. Descriptions of 25 of the 26 unprofessional behaviours were identified among 1130 (86.3%) submissions; one unprofessional behaviour (demands for sexual favours) did not feature in any Reflection submission. The three most frequently described behaviours were being spoken to rudely (53.8%, N=705), being humiliated or ridiculed (26.4%, N=346), and opinions being ignored (24.6%, N=322). Sexual assault and physical assault were described in one and nine submissions, respectively. Illustrative examples of the three most frequently described behaviours are provided in table 4.

**Table 3:** Summary of unprofessional behaviours in Reflection submissions (n=1310)

Unprofessional behaviour*	No (%) of reports describing each behaviour†
Being spoken to rudely	705 (53.8)
Being humiliated or ridiculed	346 (26.4)
Opinions being ignored	322 (24.6)
Shouted at or being the target of anger	291 (22.2)
Having unjustified allegations made	142 (10.8)
Someone withholding information which affects work performance	139 (10.6)
Being given unreasonable workloads/deadlines/tasks	137 (10.5)
Physically intimidating behaviours	85 (6.5)
Excessive monitoring of work	84 (6.4)
Repeated reminders of errors or mistakes	70 (5.3)
Being ignored or excluded	57 (4.4)
Negative comments or offensive jokes—discriminatory	43 (3.3)
Being the subject of excessive teasing/sarcasm	38 (2.9)
Treated unfairly—discriminatory	35 (2.7)
Graphic intrusive comments/questions/insinuations	19 (1.5)
Having key areas of responsibility removed or replaced with meaningless or unpleasant tasks	17 (1.3)
Being told sexually explicit or offensive jokes/comments at work	11 (0.84)
Hints or signals from others to quit your job	9 (0.69)
Physical assault	9 (0.69)
Inappropriate or unwanted touching	8 (0.61)
Threats of violence/physical abuse	5 (0.38)
Unwelcome sexual flirtations/persistent requests for dates	2 (0.15)
Being shown sexually suggestive media	2 (0.15)
Unwelcome practical jokes	1 (0.08)
Sexual assault	1 (0.08)
Demands for sexual favours	0 (0.00)
Other (eg, lack of patient centred care, failure to perform duties to a professional standard)	180 (13.7)

*Twenty-six unprofessional behaviours that appear in the LION survey.^41^ Submissions that did not describe any of the 26 behaviours were classified as ‘other.’

†More than one behaviour may be described in a submission.

**Table 4:** Illustrative examples of the three most frequently described unprofessional behaviours in Ethos submissions for Reflection

Frequently reported unprofessional behaviours	No of reports (%)	Illustrative examples		
		Event description	Profession of negative agent	Profession of submitter
Being spoken to rudely	705 (53.8)	“(deleted) was rude, abrupt and curt when the (deleted) advised he had arranged training for the (deleted) staff group during the (deleted)…When (deleted) was asked a question by (deleted) he again spoke in a rude and abrupt manner. (deleted) often presents as dismissive, rude and irritable in the staff meetings which makes the atmosphere uncomfortable for staff.”	Nurse	Non-clinical
Being humiliated or ridiculed	346 (26.4)	“I witnessed (deleted) belittle a (deleted) repeatedly both during two surgeries and in the hallway of the operating theatre. It was done continuously and in front of many staff in order to embarrass the (deleted). It was very inappropriate, unnecessary and … is distressing for both the (deleted) involved and staff who witness it.”	Medical	Nurse
Opinions being ignored	322 (24.6)	“We reviewed a patient in a joint session (deleted) and (deleted) was very condescending to the other team members (in front of the patient)…would not let people with a different opinion to hers speak or listen to them.”	Allied health	Allied health

A total of 180 (13.7%) submissions did not include any of the 26 unprofessional behaviours and were grouped together as ‘other’. Common themes identified among these submissions included, but were not limited to, lack of patient-centred care, failure to perform duties to a professional standard, work health and safety issues, and breaches of confidentiality:

‘The person above … not working with the team. Most of the shift the person spent most of her time in the sleepover room doing her personal things whilst other staff members were working hard. … Most of the time the staff member above uses a mobile constantly while on shift. This staff member does not respect people she is working with.’

‘I was sitting in the coffee shop and [deleted] … came in…She placed her belongings onto a chair and left her patient list on top…the other colleague and herself then started discussing patients on their lists inside the coffee shop within ear shot of non-staff and myself. I am concerned about the breach of patient confidentiality.’

## Discussion

Hospital staff across clinical and non-clinical professional groups actively (14 submissions/100 staff) utilised the Ethos messaging system to speak up about both positive and negative coworker behaviours. Behaviours that related to communication, collaboration and teamwork were most prevalent among submissions (both positive and negative), highlighting the importance staff place on these behaviours and their perceived role in a positive workplace culture and the delivery of quality and safe patient care.

A whole-of-hospital approach to culture change, that includes staff from all professional groups, and which provides the opportunity for staff to report both positive and negative behaviours, represents key differences in the SVHA Ethos programme compared with other widely adopted approaches that have focused primarily on addressing unprofessional behaviour among physicians.^19 24 59^ Recent studies have reported the feasibility of adapting and modifying the Vanderbilt CORS tool for reporting unprofessional behaviour among nurses^26^ and the development of a similar reporting system for medical trainees at an academic medical centre.^60^ Our results indicate that, considering the high level of cross-professional communication and collaboration, a whole-of-hospital coworker reporting system, purposely designed for use by all professional groups, represents a more efficient, sustainable and effective organisational strategy for culture change and will be used by staff.^19^

Nurses used the Ethos messaging system more than other professional groups (20.14 submissions/100 nurses) and were the most frequent subject of Recognition submissions (7.6/100 staff). While all professional groups made more positive submissions about coworkers within their own professional groups, 44.7% of Recognition submissions made about nurses were interprofessional. Patterns of interprofessional and intraprofessional reporting may in part reflect the frequent daily interactions of certain professional groups and the composition of care teams.^61^ Nurses account for approximately 50% of the healthcare workforce^62^ and are often responsible for the coordination of collaborative, interdisciplinary patient care.^63^ Medical staff were the most frequent subjects of Reflection submissions, consistent with previous reports of higher rates of unprofessional behaviour among medical staff,^13 24 64–66^ although nurses have also been identified as common source of unprofessional behaviours in hospital settings.^66 67^ Non-clinical services staff were the least frequent users and submitted twice as many reflection messages compared with recognition messages. Several factors may influence staff use of the online messaging system, including awareness of the Ethos programme, belief in the effectiveness of the programme, access to computers and opportunities at work to make online submissions, and fear that anonymity may be compromised.^19 36^

While the focus of the Ethos programme is to provide an opportunity to speak up about unprofessional behaviours, particularly those that may impact safe care, Recognition submissions describing positive behaviours accounted for almost half of all Ethos submissions. This finding highlights the value staff place on providing positive feedback and that they will invest their time in reporting these behaviours. Other studies have found online coworker recognition programmes are used more frequently than more formal Human Resource approaches,^23^ are supported by staff^2 33^ and are associated with a range of positive outcomes, including improved work performance, job satisfaction and well-being.^31 68 69^ Meaningful recognition has also been shown to be a significant predictor of decreased burnout and increased compassion satisfaction among nurses.^3^ The use of Recognition submissions at scale across professional groups is thus likely to make a positive contribution to improved organisational culture.

Providing an opportunity to recognise positive behaviours, in parallel with calling out unprofessional behaviours, signals an organisational culture that values its workforce. Perceptions of organisational cultures that support and value staff can positively impact job performance of healthcare professionals.^70^ The literatures on interventions for addressing unprofessional behaviours and staff recognition are largely siloed. Our results suggest that consideration of interventions which address both these dimensions of behaviour in tandem may be beneficial and will be supported by staff. For example, our survey of staff 2.5–3 years after Ethos implementation at five hospitals demonstrated significant reductions in staff reports of unprofessional behaviours, along with improved attitudes towards speaking up.^40^

The three most prevalent unprofessional behaviours identified in Reflection submissions were ‘being spoken to rudely’, ‘being humiliated or ridiculed’ and ‘opinions being ignored’, all behaviours frequently reported in surveys of hospital staff experiences of unprofessional behaviours.^25 41^ These findings are also consistent with a content analysis of 120 co-worker reports submitted through the Vanderbilt University Medical Center CORS system^24^ which estimated that 60% of reports described disrespectful or offensive communication.^25^

We compared the 10 most prevalent unprofessional behaviours in Reflection submissions with the 10 most frequently experienced unprofessional behaviours reported in the LION survey (completed by 5189 staff prior to Ethos implementation^41^). ‘Being spoken to rudely’ and ‘having opinions ignored’ were the two most frequent behaviours for both, and 8 of the 10 behaviours were common to both data sources. Thus, behaviours experienced by staff which motivate them to complete an Ethos Reflection submission, are largely the same unprofessional behaviours staff report when asked by survey. As such our findings lend support to the validity of staff surveys in reflecting the nature and prevalence of unprofessional behaviours in hospital settings.

Creating a safe environment where staff can raise concerns is key to safe care delivery.^71 72^ Internationally, a range of programmes seek to promote effective and assertive speaking up skills.^73 74^ A key component of the SVHA Ethos programme is capability training for staff in speaking up.^19^ Prior to the introduction of Ethos, staff with greater self-reported speaking up skills reported lower rates of frequent incivility and bullying and reduced negative impacts of unprofessional behaviour on their well-being.^41^ A survey of staff at five hospitals post Ethos found staff reported significant improvements in perceived speaking up skills.^40^ We did not examine whether there was a correlation between online reporting via Ethos and speaking up in real time. Rather, we contend that multiple components of Ethos may improve workplace culture and reduce the occurrence of unprofessional behaviour. Speaking up in the moment is often reinforced as preferable and indicative of a psychologically safe environment. Some Ethos reports indicated that staff spoke up in the moment, but also submitted a reflection report, which may indicate that events were not always resolved satisfactorily, or that the submission system was seen as a way of holding individuals to account. Concerns have been raised by others^75^ that these types of reporting systems may have unintended consequences, for example, where individuals make vexatious submissions. The role of trained staff to appropriately triage online submissions and for peer messengers to deliver effective non-punitive feedback is, therefore, crucial for perceptions of fairness and acceptability among staff and the effective delivery of the programme.

Limitations of the study included that all submissions were coded objectively based on the descriptions of events as they were provided by the authors of the submissions and could not account for any inaccurate reporting, author bias or subjectivity. We were also not able to identify submissions relating to the same individual as all data were deidentified.

## Conclusions

This study provides new information about the types of positive and unprofessional behaviours that occur both within and across professional groups within hospitals. The frequent interactions between hospital staff were also illustrated with all professional groups engaging in submissions across professional boundaries. These findings support the adoption of whole-of-hospital strategies for culture change, rather than focusing on single professional groups. The findings demonstrate that staff will equally embrace opportunities to acknowledge positive behaviours by coworkers as well as report behaviours perceived as unprofessional and which compromise patient safety. The unprofessional behaviours reported in the online messaging system mirrored those found to be reported in large-scale staff surveys, suggesting that such surveys provide a valid approach to assessing the prevalence of these behaviours and changes following intervention implementation.

## Data Availability

No data are available. Data cannot be shared for ethical/privacy reasons. The data underlying this article cannot be shared publicly due to the human research ethics committee conditions to maintain the privacy of staff who completed reports.
