# Supplementary material for: Hospital staff reports of coworker positive and unprofessional behaviours across eight hospitals: who reports what about whom?
Source: BMJ Open Qual. 2023 Nov 14;12(4):e002413. doi: 10.1136/bmjoq-2023-002413 (PMC10649603; doi:10.1136/bmjoq-2023-002413)
Supplement: Supplementary data [file bmjoq-2023-002413supp001.pdf]

Supplementary Table 1. Patterns of inter- and intra-professional reporting among Ethos Recognition submissions

			Subject of the Recognition submission:				
		**Total submissions**	**Nursing**	**Medical**	**Allied Health & Clinical Services**	**Non-clinical services**	**Management & Administrative**
**Submission made by:**	**Nursing**	787	493 (62.6%)	126 (16.0%)	57 (7.2%)	80 (10.2%)	31 (3.9%)
	**Medical**	99	39 (39.4%)	50 (50.5%)	8 (8.1%)	0 (0%)	2 (2.0%)
	**Allied Health & Clinical Services**	169	37 (21.9%)	16 (9.5%)	70 (41.4%)	40 (23.7%)	6 (3.6%)
	**Non-clinical services**	48	10 (20.8%)	2 (4.2%)	5 (10.4%)	26 (54.2%)	5 (10.4%)
	**Management & Administrative**	87	14 (16.1%)	6 (6.9%)	8 (9.2%)	29 (33.3%)	30 (34.5%)

Supplementary Table 2. Patterns of inter- and intra-professional reporting among Ethos Reflection submissions

			Subject of the Reflection submission:				
		**Total submissions**	**Nursing**	**Medical**	**Allied Health & Clinical Services**	**Non-clinical services**	**Management & Administrative**
**Submission made by:**	**Nursing**	811	439 (54.2%)	246 (30.4%)	50 (6.2%)	50 (6.2%)	25 (3.1%)
	**Medical**	119	24 (20.2%)	75 (63.0%)	4 (3.4%)	7 (5.9%)	9 (7.6%)
	**Allied Health & Clinical Services**	170	35 (20.6%)	33 (19.4%)	75 (44.1%)	9 (5.3%)	18 (10.6%)
	**Non-clinical services**	101	23 (22.8%)	1 (1.0%)	5 (5.0%)	59 (58.4%)	13 (12.9%)
	**Management & Administrative**	128	20 (15.6%)	19 (14.8%)	7 (5.5%)	8 (6.3%)	74 (57.4%)
